# Supplementary material for: SNP rs9364554 Modulates Androgen Receptor Binding and Drug Response in Prostate Cancer
Source: Biomolecules. 2025 Jan 4;15(1):64. doi: 10.3390/biom15010064 (PMC11763896; doi:10.3390/biom15010064)
Supplement: Supplementary file 1 [file biomolecules-15-00064-s001.zip › biomolecules-3359070-supplementary.pdf]

SNP rs9364554 modulates androgen receptor binding and drug response in prostate cancer

Yuqian Yan, Lei Shi, Tao Ma, Liguo Wang, Haojie Huang

Supplementary Figure S1

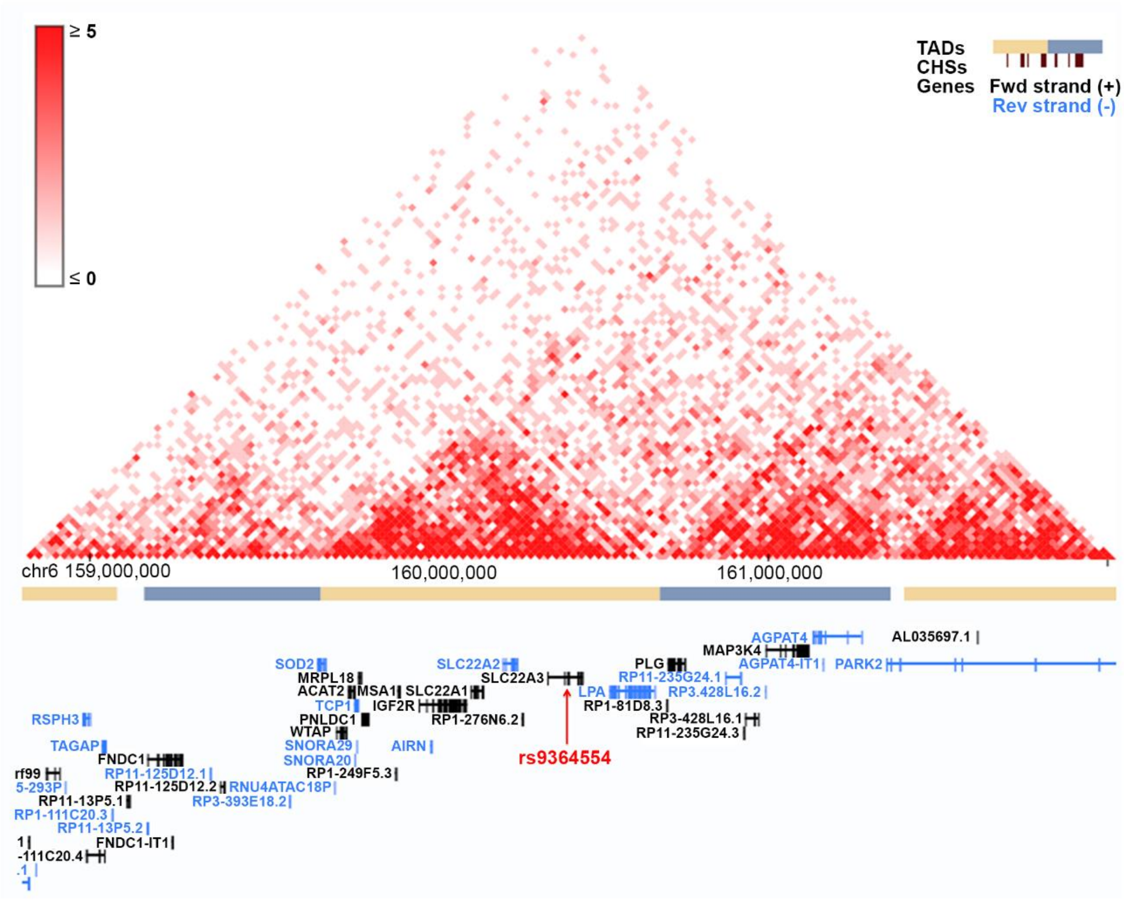

Figure S1. The SNP rs9364554 resides within a topologically associating domain (TAD). The SNP rs9364554 was identified within *SLC22A3* gene, which resides in a TAD along with other 13 transcribed genes and a long non-coding RNA (lncRNA) gene *AIRN*.

Supplementary Figure S2

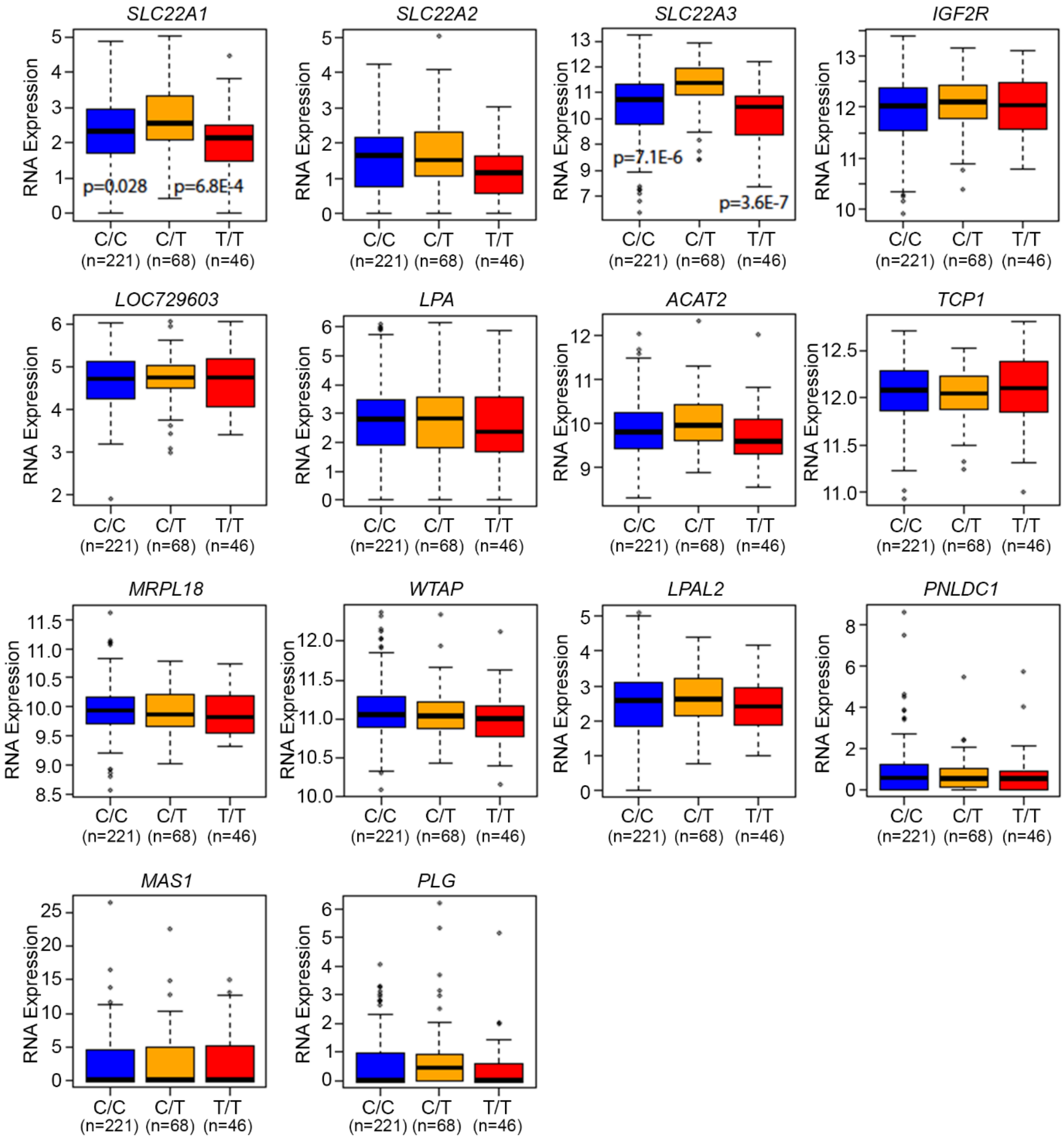

Figure S2. The transcription levels of the genes within the same TAD are analyzed according to SNP rs9364554 genotypes. The TCGA dataset [1] including 335 prostate cancer patients was used to analyze the transcription levels of 14 genes withing the same TAD.

Supplementary S3. Original films for WB and EMSA

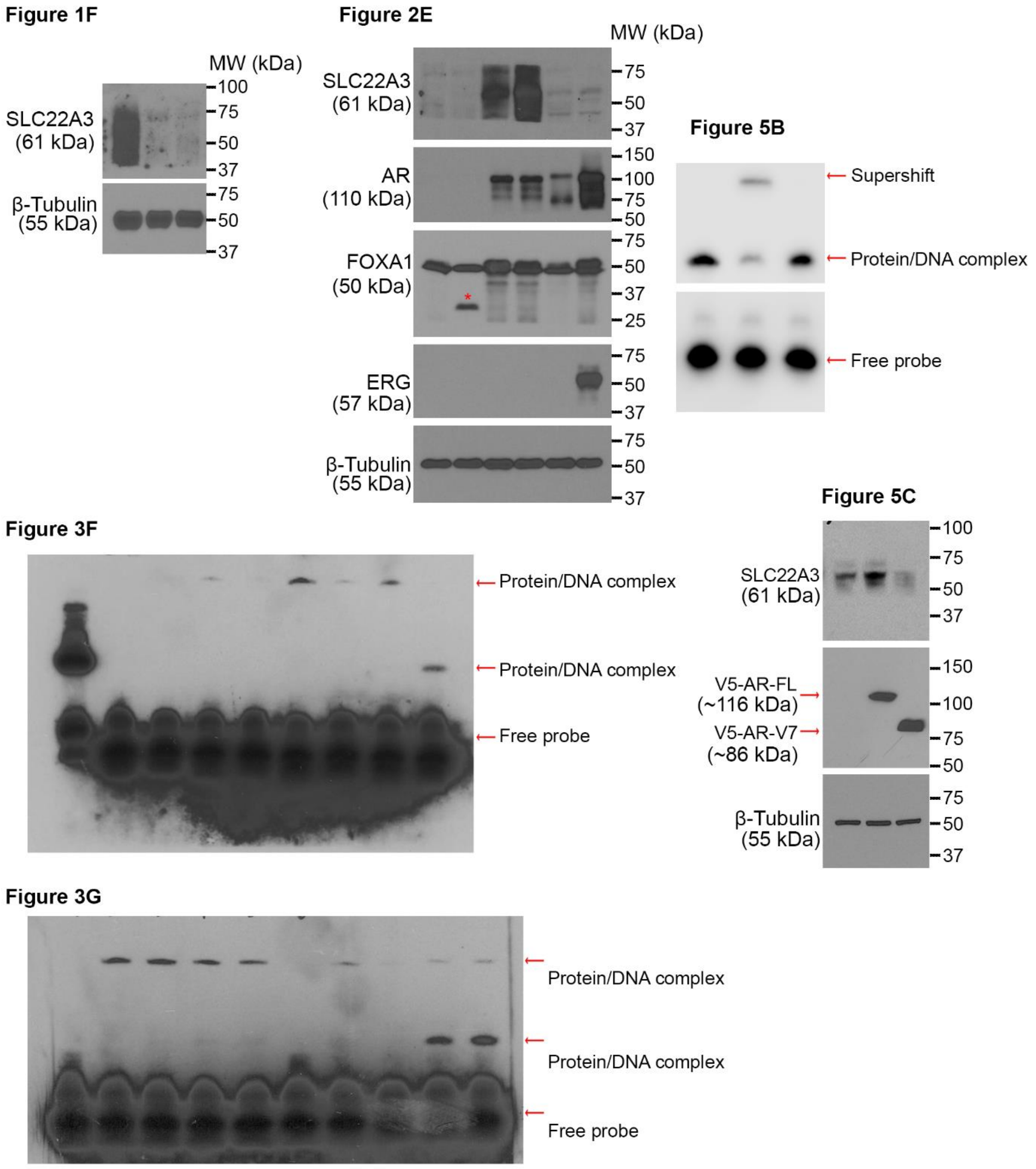

Figure S3. The original WB and EMSA films related to main figures (Figures 1–3 and 5).

Table S1. Sequences of gene-specific siRNAs

siRNA	Sequence (5' to 3')	Company and Cat. No
siNS	UAGCGACUAAACACAUCAA	Dharmacon; D-001206-13-05
siAR	CGUGCAGCCUAUUGCGAGAUU	Dharmacon; D-003400-18-0002
siERG	*GACAUCCUUCUCUCACAU*	Dharmacon; D-001210-01
siFOXA1	GCACUGCAAUACUCGCCUU	Horizon Discovery; M-010319-01

Table S2. Oligonucleotides

Gene	Usage	Forward (5'-3')	Reverse (5'-3')	Species
GAPDH	RT-qPCR	ACCCACTCCTCCACCTTT GAC	TGTTGCTGTAGCCAAAT TCGTT	Human
SLC22A3	RT-qPCR	GCTTCCTGACTGGAGCAT TC	AGGAAGCGGAAGATCA CAAA	Human
SLC22A3	*Sequencing*	CCCTTGAAGCTTGGTCAT GT	TCAAAACCTTGGACTTA CCTCTG	Human
SNP C allele	*EMSA*	CCTTGTCACTCTTTGAGTG AACACA	TGTGTTCACTCAAAGAG TGACAAGG	Human
SNP T allele	*EMSA*	CCTTGTCACTTTTTGAGTG AACACA	TGTGTTCACTCAAAGAG TGACAAGG	Human
TCP1	*ChIP-qPCR*	GCCAATTTGGAAGGTGAA GA	AATCATTTGCCCCACGT AAG	Human
SLC22A3	*ChIP-qPCR*	GGTTTACAGATCATAAAA TGTGTGG	ATGAGTGCAAGCTGGGA AAC	Human
SLC22A3	*ChIP-qPCR*	GGTTTACAGATCATAAAA TGTGTGG	ATGAGTGCAAGCTGGGA AAC	Human
SLC22A3	*ChIP-qPCR*	GGTTTACAGATCATAAAA TGTGTGG	ATGAGTGCAAGCTGGGA AAC	Human
SLC22A3 sgRNA-#1	sgRNA clone	CACCGGGGTGGCGCGGG GGAGGCGG	AAACCCGCCTCCCCCGC GCCACCC	Human
SLC22A3 sgRNA-#2	sgRNA clone	CACCGAGAGAGGCGGGG GCCGCGGG	AAACCCCGCGGCCCCCG CCTCTCTC	Human
IGF2R	RT-qPCR	GGTGCCATGCTATGTGTT TG	AAACGCCTGGTGTCCTC TTA	Human

Table S3. Information for bacterium strains and virus packaging vectors

**Virus strains and recombinant DNA**		
**Name**	**Company**	**Cat. No**
E. coli DH5α	Thermo Fisher	Cat#18258012
psPAX2	Addgene	Cat# 12260
pMD2.G	Addgene	Cat# 12259

1. Cancer Genome Atlas Research, N., *The Molecular Taxonomy of Primary Prostate Cancer*. Cell, 2015. **163**(4): p. 1011-25.
